# Effect of gluten-free diet on autoimmune thyroiditis progression in patients with no symptoms or histology of celiac disease: a meta-analysis

**DOI:** 10.3389/fendo.2023.1200372

**Published:** 2023-07-24

**Authors:** Tommaso Piticchio, Francesco Frasca, Pasqualino Malandrino, Pierpaolo Trimboli, Nunzia Carrubba, Andrea Tumminia, Federica Vinciguerra, Lucia Frittitta

**Affiliations:** ^1^ Endocrinology Section, Department of Clinical and Experimental Medicine, University of Catania, Catania, Italy; ^2^ Endocrinology Unit, Garibaldi Hospital, Catania, Italy; ^3^ Servizio di Endocrinologia e Diabetologia, Ospedale Regionale di Lugano, Ente Ospedaliero Cantonale (EOC), Lugano, Switzerland; ^4^ Facoltà di Scienze Biomediche, Università della Svizzera Italiana (USI), Lugano, Switzerland; ^5^ Diabetes, Obesity and Dietetic Center, Garibaldi Hospital, Catania, Italy

**Keywords:** gluten-free diet, autoimmune thyroiditis, thyroid, anti-thyroglobulin (TgAb), anti-thyroperoxidase (TPOAb), TSH, FT4, FT3

## Abstract

**Background:**

Hashimoto’s thyroiditis (HT) is the most common autoimmune disease. HT may be associated with nonthyroidal autoimmune diseases, including celiac disease (CD) or other gluten-related conditions (GRC). In the last years, interest about gluten-free diet (GFD) has increased for its supposed extraintestinal anti-inflammatory effect; thus, many patients with HT initiate GFD on their own.

**Objectives:**

The aim of this meta-analysis is to examine all available data in literature about the effect of a GFD on TgAb, TPOAb, TSH, FT4, and FT3 levels in patients with HT and no symptoms or histology of CD.

**Methods:**

The study was conducted according to MOOSE (Meta-analysis Of Observational Studies in Epidemiology). The search was performed on databases PubMed and Scopus. The last search was performed on 7 February 2023. Quality assessment was performed. Meta-analyses were performed using the random-effect model. Hedges’ *g* was used to measure the effect size (ES). Statistical analyses were performed using StataSE 17.

**Results:**

The online search retrieved 409 articles, and 4 studies with a total of 87 patients were finally included for quantitative analysis. The risk of bias was generally low. The mean period of GFD was almost 6 months. The meta-analyses showed reduction in antibody levels with ES: −0.39 for TgAb (95% CI: −0.81 to +0.02; *p* = 0.06; *I*² = 46.98%) and −0.40 for TPOAb (95% CI: −0.82 to +0.03; *p* = 0.07; *I*² = 47.58%). TSH showed a reduction with ES: −0.35 (95% CI: −0.64 to −0.05; *p* = 0.02; *I*² = 0%) and FT4 showed an increase with ES: +0.35% (95% CI: 0.06 to 0.64; *p* = 0.02; *I*² = 0%). FT3 did not display variations (ES: 0.05; 95% CI: −0.38 to +0.48; *p* = 0.82; *I*² = 51%). The heterogeneity of TgAb, TPOAb, and FT3 data was solved performing sub-analyses between patients with or without GRC (TgAb *p* = 0.02; TPOAb *p* = 0.02; FT3 *p* = 0.04) and only for FT3, performing a sub-analysis between patients taking and not taking LT4 (*p* = 0.03).

**Conclusion:**

This is the first meta-analysis investigating the effect of GFD on HT. Our results seem to indicate a positive effect of the gluten deprivation on thyroid function and its inflammation, particularly in patients with HT and GRC. However, current lines of evidence are not yet sufficient to recommend this dietary approach to all patients with a diagnosis of HT.

## 1 Introduction

Hashimoto’s thyroiditis (HT) is the most common autoimmune disease worldwide.

HT is caused by a lymphocyte infiltration within the thyroid gland and the subsequent production of antibodies against thyroglobulin (TgAb) and thyroid peroxidase (TPOAb). HT is present in approximately 5% of the general population, with a female predominance (F:M ratio >10:1), and it is the most common cause of hypothyroidism in developed countries (1, 2).

HT may be associated with several nonthyroidal autoimmune diseases (3–5).

One of the most frequent associations is with gastrointestinal autoimmune disorders, including celiac disease (CD) and gluten-related conditions (GRC) (i.e., non-celiac gluten/wheat sensitivity—NCGS; incidental finding of positive anti-tissue transglutaminase antibodies—anti-tTG—without clinical symptoms or histological confirmation of CD), whose frequency is elevated in patients with HT (2%–9% of overall HT patients) (4, 6–8). In turn, autoimmune thyroiditis is the predominant autoimmune disorder coexisting in patients with CD (8–10) and the most frequently reported NCGS-associated autoimmune disorder in a study (11). The latter finding was indirectly confirmed also by an Italian experience (12), showing that autoimmune thyroiditis could be a risk factor for the evolution towards NCGS in a group of patients with minimal duodenal inflammation (13).

Moreover, the frequent overlap between HT and CD was explained by authors through the identification of common etiopathogenetic triggers including sharing of similar susceptible HLA haplotypes (14, 15) and gut microbiota dysbiosis. Dysbiosis seems to increase gut permeability, allowing antigen permeation with activation of the immune system and cross-reaction with extraintestinal tissues (16).

Gluten-free diet (GFD) is the treatment par excellence of CD, so it is considered a necessary therapeutic intervention for all celiac patients.

However, it may display a general anti-inflammatory effect also in extraintestinal autoimmune inflammatory diseases, thus improving symptoms and being adjuvant to conventional drug therapies (17, 18). Hence, it is reasonable to suppose that GFD may also display a favorable effect on thyroid autoimmunity. Several mechanisms may explain the potential beneficial effect of GFD on HT, including concomitant reduction of circulating levels of proinflammatory cytokines (19, 20) and decrease of gut permeability (16).

Furthermore, adherence to GFD may positively influence the absorption of selenium and vitamin D essential elements for thyroid function and health (21–23).

Vitamin D insufficiency, indeed, has been linked to several autoimmune disorders and an inverse relationship between vitamin D levels and ATPO titer has been also reported (24).

Finally, it has been demonstrated that in hypothyroid HT patients, the increased requirement of levothyroxine dose pro/kg/day may be reverted following the GFD for a likely improvement in intestinal absorption (25).

Although GFD is mainly recommended in CD, many people worldwide actually follow a GFD on their own due to its supposed anti-inflammatory effects even in the absence of a concomitant CD. However, the current clinical lines of evidence regarding the effect of GFD on HT in patients are still scanty and inconclusive. Hence, the aim of this study is to review and meta-analyze all quantitative data currently available in literature about the effect of GFD on anti-thyroid antibody and thyroid hormone levels in patients with HT and no symptoms or histology of CD.

## 2 Materials and methods

### 2.1 Construction of the review

The systematic review was performed according to MOOSE (Meta-analysis Of Observational Studies in Epidemiology) (**Supplementary Table 1**) (26).

### 2.2 Data sources and searches

The search was conducted on the online databases PubMed and Scopus by two independent authors (TP and FF) and references of included studies were screened to find further papers. Terms used to perform the search were as follows: (“Gluten-free diet” or “Gluten deprivation” or “Gluten exclusion”) and (“Thyroiditis” and/or “Hashimoto” and/or “TSH” and/or “FT4” and/or “Anti-Thyroid Antibodies” and/or “TgAb” and/or “TPOAb”). No publication-year restriction was applied, and only papers in English were considered.

We included all studies with original data and review articles were considered for additional papers that could be missing from our literature search.

The last search was performed on 7 February 2023.

The two investigators independently searched papers, screened both titles and abstracts, reviewed the full texts, and selected articles for their inclusion.

Data were cross-checked, while the discrepancies were resolved after discussion between the authors.

### 2.3 Study selection

Inclusion criteria were as follows: studies reporting (a) data of TSH and/or FT4, and/or FT3, and/or TgAb, and/or TPOAb of adult HT patients at baseline and after a period of monitored GFD; and (b) patients without clinical symptoms of CD (including also adult patients with an incidental finding of positive anti-tTG) or with negative intestinal histology for CD (e.g., patients diagnosed with NCGS).

Exclusion criteria were as follows: studies (a) with patients who started therapy with levothyroxine (LT4) at the same time as GFD; (b) with patients who changed LT4 dosage during the GFD period; (c) with patients diagnosed with CD; (d) with unclear data; and (e) with overlapping data.

When raw data, necessary for our study, were not available in the paper text, the corresponding authors were asked to provide them.

### 2.4 Data extraction

All data were obtained from the main text, tables, figures, and supplementary material of papers. The following information were independently searched and extracted by two authors (TP and FF) from the included studies: authors; year of publication; number of patients; sex of patients; mean and standard deviation (SD) of patient’s age; mean and SD of patient’s BMI; mean and SD of TSH, FT4, FT3, TgAb, and TPOAb at baseline and after a monitored GFD period; GFD period; LT4 therapy; and presence of GRC. Whenever possible, we inserted values of 6-month control in our database; when not available, the closest control was included.

### 2.5 Quality of selected studies and assessment of the risk of bias

The risk of bias for included studies was assessed by two reviewers (TP and FF) through the National Heart, Lung, and Blood Institute Quality Assessment Tool for Observational Studies (27).

### 2.6 Statistical analysis

From extracted summary data, a meta-analysis was performed for response of continuous outcomes (i.e., TSH, FT4, FT3, TgAb, and TPOAb). Hedges’ *g* was used to measure the effect size (ES). The random-effects model of meta-analysis was used to incorporate heterogeneity, which was assessed by using *I^2^*. Pooled data were presented with 95% confidence intervals (95% CI). When heterogeneity was found, subgroup and meta-regression analyses were performed to explore the causes using several covariates (i.e., sample size, continent, duration of GFD, mean age, mean BMI, LT4 therapy, and GRC patients included). A *p* < 0.05 was regarded as significant. All calculations were performed using STATA/SE 17.0.

## 3 Results

### 3.1 Studies retrieved

**Figure 1:**
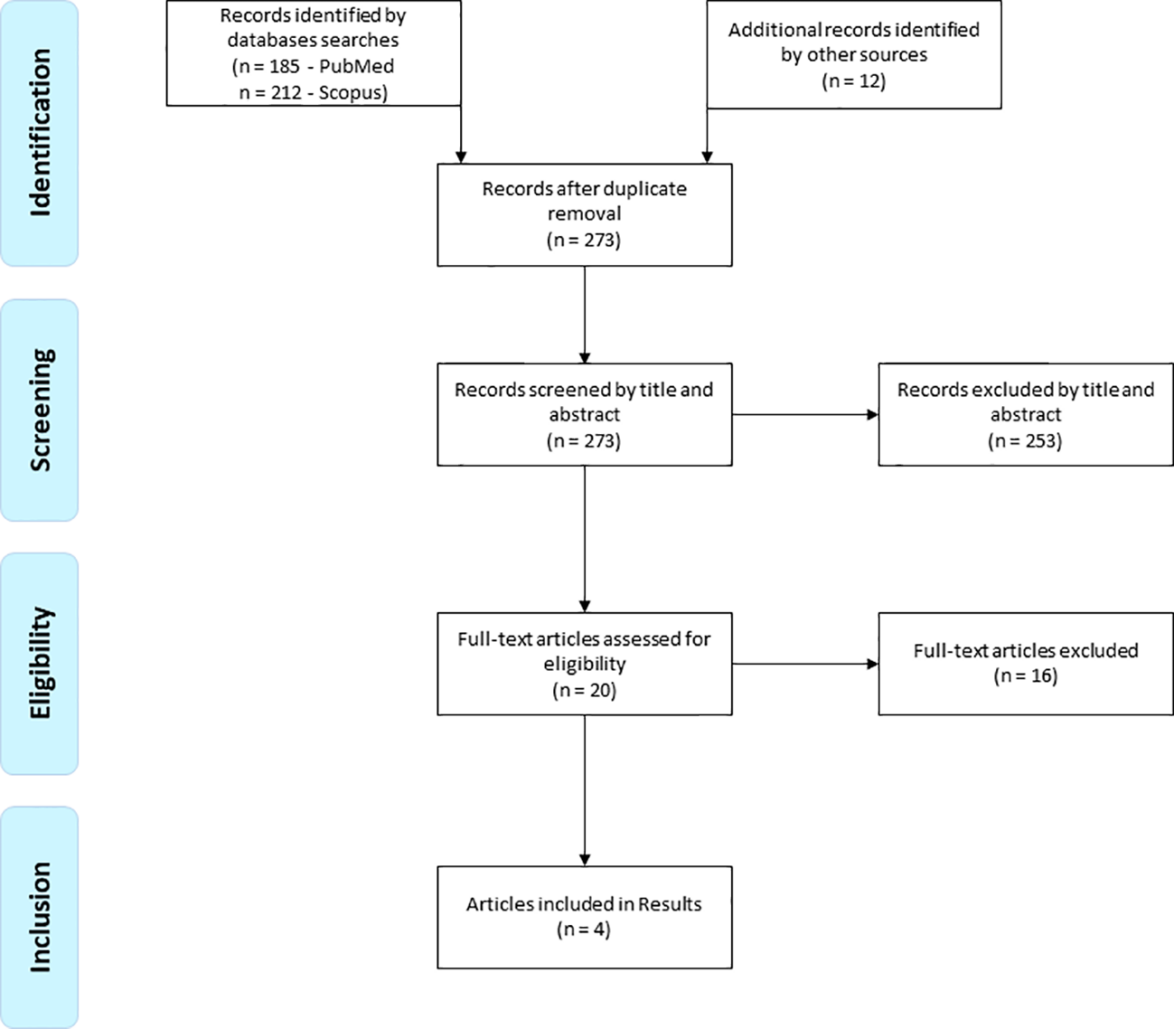
Flow of records found.

A total of 409 records were initially found by the above search strategy. After duplicate removal and screening by title and abstract, 20 papers were selected for retrieving their full text. Finally, four studies were included in the systematic review (6, 28–30) (**Figure 1**).

### 3.2 Study quality assessment

The risk of bias of the included studies is shown in the **Supplementary Material**(**Supplementary Table 2**). Overall, 12 of 14 items could be judged as low in all studies. Time frame of exposure (item 7) was assessed at high risk of bias in one study because of the narrow period of gluten exclusion (i.e., ≅3 months). No studies reported information about power or sample size justification.

### 3.3 Qualitative analysis (systematic review)

The papers included in the present systematic review were published between 2019 and 2022. All of them were prospective cohort studies. Three studies have been carried out by European institutes and one by a US institute. Two studies included patients with GRC: the first one included adults with diagnosis of NCGS (28) and the second one included adults with an incidental finding of anti-tTG antibodies in the absence of CD symptoms (30). The total number of HT patients who underwent GFD was 87, 47 of whom have GRC. All patients were female. Patient ages ranged from 25 to 42 years. Patient BMI ranged from 19.5 to 30 kg/m^2^. The mean time frame of gluten exclusion was of 5.5 months. All studies excluded subjects with hyperthyroidism or other endocrine disorders, impaired renal or hepatic function, heart diseases, acute inflammatory processes, and pregnancy or lactation. The main characteristics of included studies are summarized in **Table 1**.

**Table 1:** Main characteristics of included studies.

Ref.	Authors	Year	Geographic area	Patients	Age (mean)	BMI (mean)	Follow-up (months)	GRC
(28)	Krysiak et al.	2022	Europe	31	35	22.6	6	Yes
(6)	Pobłocki et al.	2021	Europe	28	36.6	26.1	6	No
(29)	Abbott et al.	2019	North America	12	35.6	24.9	3	No
(30)	Krysiak et al.	2019	Europe	16	30	22.9	6	Yes

### 3.4 Quantitative analysis (meta‐analysis)

**Figure 2:**
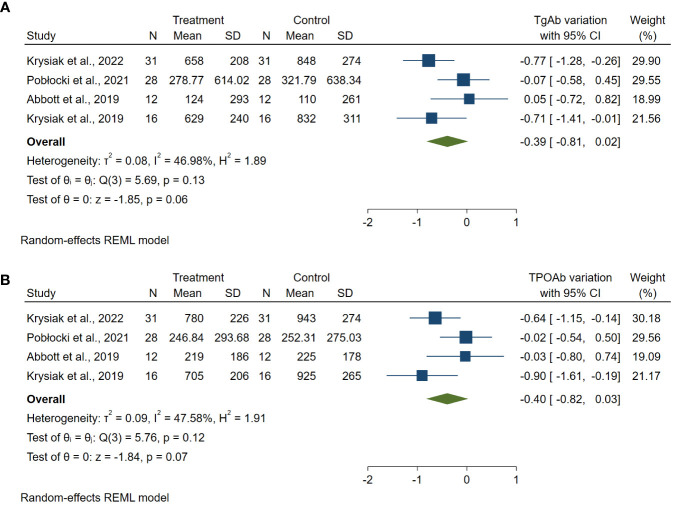
Forest plot TgAb **(A)** and TPOAb **(B)** variations. Legend: Any square identifies the weight of the study. The diamond represents the pooled result and its wideness indicates 95% CI.

The meta-analyses of variations in thyroid antibody levels after a gluten deprivation period showed an overall reduction trend in antibody levels with ES: −0.39 for TgAb (95% CI: −0.81 to +0.02; *p* = 0.06; *I*² = 46.98%) (**Figure 2A**) and −0.40 for TPOAb (95% CI: −0.82 to +0.03; *p* = 0.07; *I*² = 47.58%) (**Figure 2B**).

**Figure 3:**
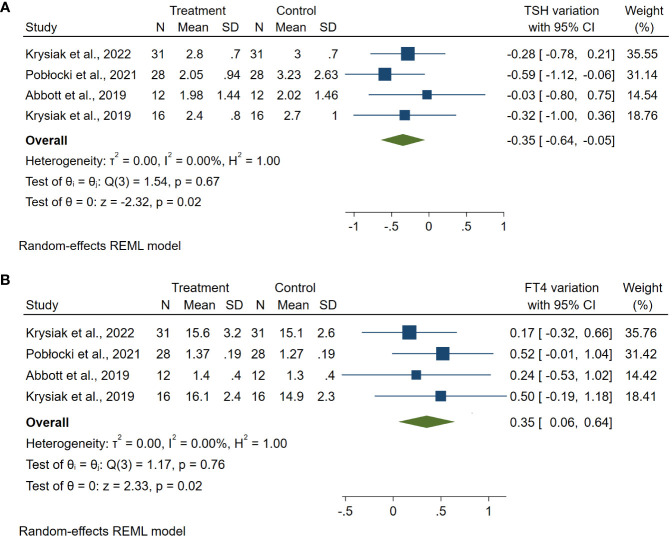
Forest plot TSH **(A)** and FT4 **(B)** variations. Legend: Any square identifies the weight of the study. The diamond represents the pooled result and its wideness indicates 95% CI.

The meta-analyses of changes in TSH and FT4 after a gluten deprivation period showed an overall reduction trend in TSH levels with ES: −0.35 (95% CI: −0.64 to −0.05; *p* = 0.02; *I*² = 0%) (**Figure 3A**) and an overall increasing trend in FT4 levels with ES: +0.35% (95% CI: 0.06 to 0.64; *p* = 0.02; *I*² = 0%) (**Figure 3B**).

Finally, meta-analysis of FT3 levels did not display any substantial variations compared to the pre-diet levels (ES: 0.05; 95% CI: −0.38 to +0.48; *p* = 0.82; *I*² = 51%) (**Supplementary Figure 1**).

**Figure 4:**
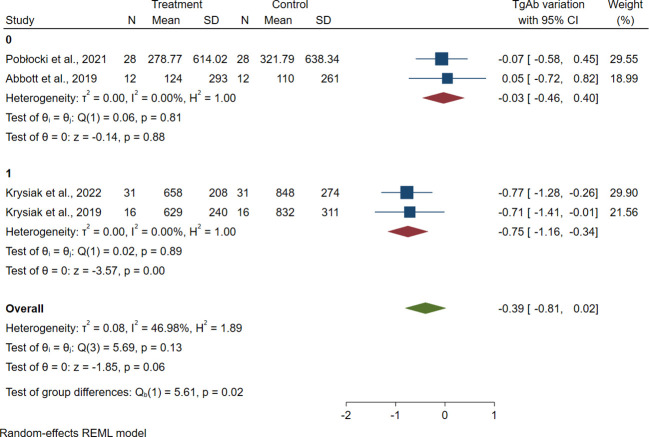
Forest plot TgAb sub-analysis. Legend: Any square identifies the weight of the study. The diamond represents the pooled result and its wideness indicates 95% CI. 0: Studies including patients with HT. 1: Studies including patients with HT and GRC.

**Figure 5:**
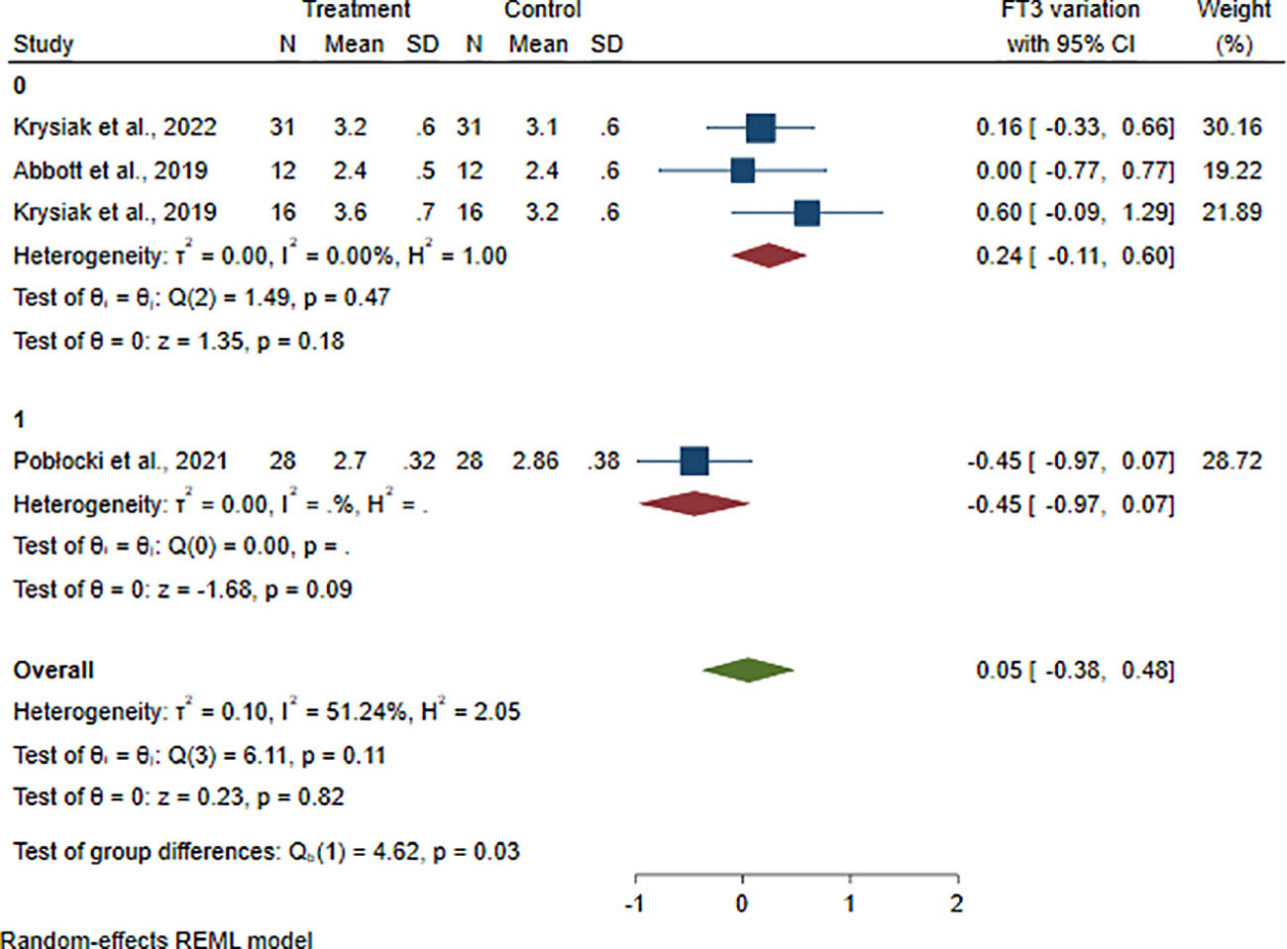
Forest plot FT3 sub-analysis. Legend: Any square identifies the weight of the study. The diamond represents the pooled result and its wideness indicates 95% CI. 0: Studies including patients not taking LT4. 1: Studies including patients taking LT4 (stable dose already taken before dietary intervention).

The heterogeneity of meta-analyses was explored according to the above covariates when appropriate. The heterogeneity of TgAb, TPOAb, and FT3 meta-analyses was solved performing sub-analyses between patients with or without GRC (TgAb *p* = 0.02; TPOAb *p* = 0.02; FT3 *p* = 0.04) (**Figure 4**, **Supplementary Figures 2**, **3**). The heterogeneity of FT3 meta-analysis was also solved performing a sub-analysis between patients taking LT4 (stable dose taken already before the dietary intervention) and those not taking it (*p* = 0.03) (**Figure 5**).

## 4 Discussion

The present meta-analysis was designed to calculate the effect of gluten deprivation on thyroid antibody and hormone levels in subjects with chronic autoimmune thyroiditis including all the data currently available in the literature.

HT is the most common autoimmune disease worldwide and is characterized by a high prevalence in female patients and frequent thyroid functional impairment (1, 2). Thyroiditis, in a percentage variable between 14% and 29% of cases, may be associated with further endocrine and non-endocrine autoimmune diseases (3–5). The most frequent associations involve rheumatic and gastrointestinal autoimmune diseases (6, 8, 17).

Therefore, data emerging from the present meta-analysis on GFD in HT patients may have a relevant impact on clinical practice.

In accordance with the epidemiologic data regarding a higher prevalence of autoimmune thyroid disease in women than in men, the recruited papers analyzed adult women between 25 and 42 years.

A mean period of almost 6 months of GFD induced a steady decrease in TgAb and TPOAb titers, which nearly reached statistical significance (**Figure 2**).

When the heterogeneity was explored, it has been found that the reduction in antibody titers mostly affects GRC patients, with a large ES of gluten deprivation (**Figure 4**, **Supplementary Figure 2**).

Patients with HT and/or CD shared a common genetic susceptibility regarding genes encoding the major histocompatibility complex (HLA-B8, HLA-D3, HLA-DQ2, and HLA-DQ8), the cytotoxic T-lymphocyte-associated antigen-4 (CTLA-4), IL-18, and IFN-γ (14).

This common genetic susceptibility could also be shared with the emerging forms of GRC, about which there are few data in the literature as they were often un- or mis-diagnosed.

Moreover, in patients with CD or GRC and/or HT, a condition known as “leaky gut” has been described, characterized by an increased permeability of the intestinal barrier that allows the entrance of immunogenic exogen compounds into the systemic circulation (31–33).

In accordance with these lines of evidence, we can speculate that gluten deprivation in patients with HT and GRC may improve an already described mild inflammatory state of the intestine (13), thereby reducing its permeability and the consequent systemic autoimmune inflammatory triggers resulting in TgAb and TPOAb reduction.

Furthermore, it is reasonable to suppose that GFD may also improve gut microbiota composition and the dysbiotic state, which, in turn, may sustain the vicious circle of gut epithelium damage, chronic inflammation, and, in people with a genetic predisposing background, the trigger of autoimmunity (34–36).

Importantly, TSH and FT4 meta-analyses showed a statistically significant improvement of both hormones with no heterogeneity (**Figure 3**).

These relevant findings may be explained by a role of GFD in better absorption of iodine, selenium, zinc, vitamin D, and all nutrients essential for the adequate thyroid functioning and the regulation of its autoimmunity (21, 37, 38). Furthermore, in HT patients treated with levothyroxine, improved absorption of therapy should be taken into account during GFD (25).

Regarding FT3 levels, there seemed to be no significant change after the dietary intervention in our overall analysis (**Supplementary Figure 1**).

However, it is interesting to note that by performing the sub-analyses based on levothyroxine therapy or in the presence of concomitant GRC, a statistically significant difference emerges in both cases (**Supplementary Figure 3**, **Figure 5**).

We can speculate that the peripheral deiodinase activity is improved by the reduction in systemic inflammatory activity induced by the GFD and that it could be impaired by assumption of LT4 (39, 40).

In this scenario, the results of our study suggest that GFD seems to ameliorate thyroid homeostasis in patients with HT, especially if they also suffer by disorders attributable to GRC. However, the findings are currently not strong enough to apply this approach to all patients with HT.

GFD, in fact, may result in increased consumption of gluten-free processed foods that have higher levels of lipids, trans fats, salt, and glycemic index, and lower levels of protein, fiber, and micronutrients than gluten-containing products, particularly compared to whole grains (41). Therefore, if proposed on a large scale, this dietary regimen may lead to an unjustified higher long-term risk of metabolic diseases (42).

Although our systematic review included almost 100 patients with HT undergoing a period of gluten deprivation, there were some limitations. To date, the data present in the literature about the topic are still scanty. Despite the fact that all studies excluded patients with a diagnosis of CD, some of them included subjects with GRC. In particular, one study included patients with an incidental finding of positive anti-tTG antibodies without clinical symptoms of CD, and as no small bowel biopsy was performed, it is possible that some of them might have had a form of asymptomatic CD (30). However, this laboratory finding in adulthood has widely variable sensitivity and specificity (35%–100% and 0%–100%, respectively) with consequent poor diagnostic significance (43) and the most recent meta-analysis on this topic, simulating the application of the obtained figures in a cohort of adult subjects with positive anti-tTG, which showed a percentage of false positives for CD of 87% (44).

Furthermore, another paper in our study observed patients diagnosed with NCGS, but it is necessary to highlight that the latter presented negative anti-tTG titer and no villous atrophy on biopsy (28).

Finally, before the dietary intervention, some patients have already been on therapy with a stable dose of LT4, unmodified during the period examined.

These two described covariates best resolved the heterogeneity of the data collected. Therefore, these discrepancies allowed us to fully explore the heterogeneity of the pooled data and thus achieve the most robust findings with the available literature.

## 5 Conclusions

To the best of our knowledge, this is the first meta-analysis to investigate the effect of gluten deprivation on thyroid antibody and hormone levels in patients with chronic autoimmune thyroiditis and no symptoms or histology of CD.

The evidence found must be assumed with caution given the relatively small number of patients included. Our results seem to indicate a positive effect of the gluten deprivation on thyroid function and its inflammation, particularly in patients with GRC. However, current lines of evidence are not yet sufficient to recommend this diet to all non-celiac patients with HT. These findings highlight the need for new randomized trials on large patient cohorts.

## Data availability statement

The original contributions presented in the study are included in the article/**Supplementary Material**. Further inquiries can be directed to the corresponding author.

## Author contributions

Conceptualization: LF, FF, and TP. Methodology: PM, PT, and TP. Software: TP. Validation: LF, FF, PT, and PM. Formal analysis: LF, FF, and FV. Investigation: TP, FF, and NC. Data curation: TP and NC. Writing—original draft preparation: TP. Writing—review and editing: TP, LF, FF, and FV. Visualization: AT, PT, NC, FF, and LF.Supervision: LF and FF. Funding acquisition: LF.
