# Supplementary material for: Effect of gluten-free diet on autoimmune thyroiditis progression in patients with no symptoms or histology of celiac disease: a meta-analysis
Source: Front Endocrinol (Lausanne). 2023 Jul 24;14:1200372. doi: 10.3389/fendo.2023.1200372 (PMC10405818; doi:10.3389/fendo.2023.1200372)
Supplement: Supplementary file 1 [file DataSheet_1.docx]

Effect of gluten-free diet on autoimmune thyroiditis progression in patients with no symptoms or histology of celiac disease: a meta-analysis

Tommaso Piticchio^*^, Francesco Frasca, Pasqualino Malandrino, Pierpaolo Trimboli, Nunzia Carrubba, Andrea Tumminia, Federica Vinciguerra and Lucia Frittitta

*** Correspondence:**

**Corresponding Author**

tommaso.piticchio@phd.unict.it

**Supplementary material**

1. Supplementary Table 1: MOOSE (Meta-analyses Of Observational Studies in Epidemiology) Checklist.
2. Supplementary Table 2. Risk of bias summary: review of authors’ judgements about each risk of bias item for each included in the meta-analysis.
3. Supplementary Figure 1.
4. Supplementary Figure 2.
5. Supplementary Figure 3.

**Supplementary Table 1: MOOSE (Meta-analyses Of Observational Studies in Epidemiology) Checklist**

| **Item No** | **Recommendation** | **Page** |
| --- | --- | --- |
| Reporting of background should include | | |
| 1 | Problem definition | 2-3 |
| 2 | Hypothesis statement | 2-3 |
| 3 | Description of study outcome(s) | 2-3 |
| 4 | Type of exposure or intervention used | 2-3 |
| 5 | Type of study designs used | 2-3 |
| 6 | Study population | 2-3 |
| Reporting of search strategy should include | | |
| 7 | Qualifications of searchers (eg, librarians and investigators) | 3 |
| 8 | Search strategy, including time period included in the synthesis and key words | 3 |
| 9 | Effort to include all available studies, including contact with authors | 3 |
| 10 | Databases and registries searched | 3 |
| 11 | Search software used, name and version, including special features used (eg, explosion) | 3 |
| 12 | Use of hand searching (eg, reference lists of obtained articles) | 3 |
| 13 | List of citations located and those excluded, including justification | 3 |
| 14 | Method of addressing articles published in languages other than English | 3 |
| 15 | Method of handling abstracts and unpublished studies | 3 |
| 16 | Description of any contact with authors | 3 |
| Reporting of methods should include | | |
| 17 | Description of relevance or appropriateness of studies assembled for assessing the hypothesis to be tested | 3 |
| 18 | Rationale for the selection and coding of data (eg, sound clinical principles or convenience) | 3 |
| 19 | Documentation of how data were classified and coded (eg, multiple raters, blinding and interrater reliability) | 3 |
| 20 | Assessment of confounding (eg, comparability of cases and controls in studies where appropriate) | 3 |
| 21 | Assessment of study quality, including blinding of quality assessors, stratification or regression on possible predictors of study results | 3 |
| 22 | Assessment of heterogeneity | 3 |
| 23 | Description of statistical methods (eg, complete description of fixed or random effects models, justification of whether the chosen models account for predictors of study results, dose-response models, or cumulative meta-analysis) in sufficient detail to be replicated | 3 |
| 24 | Provision of appropriate tables and graphics | 3 |
| Reporting of results should include | | |
| 25 | Graphic summarizing individual study estimates and overall estimate | 3-5 |
| 26 | Table giving descriptive information for each study included | 3-5 |
| 27 | Results of sensitivity testing (eg, subgroup analysis) | 3-5 |
| 28 | Indication of statistical uncertainty of findings | 3-5 |
| Reporting of discussion should include | | |
| 29 | Quantitative assessment of bias (eg, publication bias) | 5-7 |
| 30 | Justification for exclusion (eg, exclusion of non-English language citations) | 5-7 |
| 31 | Assessment of quality of included studies | 5-7 |
| Reporting of conclusions should include | | |
| 32 | Consideration of alternative explanations for observed results | 7 |
| 33 | Generalization of the conclusions (ie, appropriate for the data presented and within the domain of the literature review) | 7 |
| 34 | Guidelines for future research | 7 |
| 35 | Disclosure of funding source | 7 |

*From*: Stroup DF, Berlin JA, Morton SC, et al, for the Meta-analysis Of Observational Studies in Epidemiology (MOOSE) Group. Meta-analysis of Observational Studies in Epidemiology. A Proposal for Reporting. *JAMA*. 2000;283(15):2008-2012. doi: 10.1001/jama.283.15.2008.

**Supplemental Table 2. Risk of bias summary: review of authors’ judgements about each risk of bias item for each study included in the meta-analysis.**

|  | **1** | **2** | **3** | **4** | **5** | **6** | **7** | **8** | **9** | **10** | **11** | **12** | **13** | **14** |
| --- | --- | --- | --- | --- | --- | --- | --- | --- | --- | --- | --- | --- | --- | --- |
| Krysiak et al., 2022 | L | L | L | L | N.R. | L | L | L | L | L | L | L | L | L |
| Pobłocki et al., 2021 | L | L | L | L | N.R. | L | L | L | L | L | L | L | L | L |
| Abbott et al., 2019 | L | L | L | L | N.R. | L | H | L | L | L | L | L | L | L |
| Krysiak et al., 2019 | L | L | L | L | N.R. | L | L | L | L | L | L | L | L | L |

L, low risk; H, high risk; N.R., not reported, N.A., not applicable

Questions:

1. Was the research question or objective in this paper clearly stated?

2. Was the study population clearly specified and defined?

3. Was the participation rate of eligible persons at least 50%?

4. Were all the subjects selected or recruited from the same or similar populations (including the same time period)? Were inclusion and exclusion criteria for being in the study prespecified and applied uniformly to all participants?

5. Was a sample size justification, power description, or variance and effect estimates provided?

6. Were the exposure(s) of interest measured prior to the outcome(s) being measured?

7. Was the timeframe sufficient so that one could reasonably expect to see an association between exposure and outcome if it existed?

8. For exposures that can vary in amount or level, did the study examine different levels of the exposure as related to the outcome (e.g., categories of exposure, or exposure measured as continuous variable)?

9. Were the exposure measures (independent variables) clearly defined, valid, reliable, and implemented consistently across all study participants?

10. Was the exposure(s) assessed more than once over time?

11. Were the outcome measures (dependent variables) clearly defined, valid, reliable, and implemented consistently across all study participants?

12. Were the outcome assessors blinded to the exposure status of participants?

13. Was loss to follow-up after baseline 20% or less?

14. Were key potential confounding variables measured and adjusted statistically for their impact on the relationship between exposure(s) and outcome(s)?

## Supplementary Figures


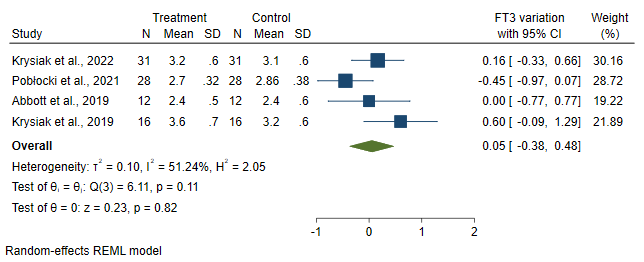
**Supplementary Figure 1.** Forest plot FT3 variations. Legend: Any square identifies the weight of the study. The diamond represents the pooled result and its wideness indicates 95%CI.


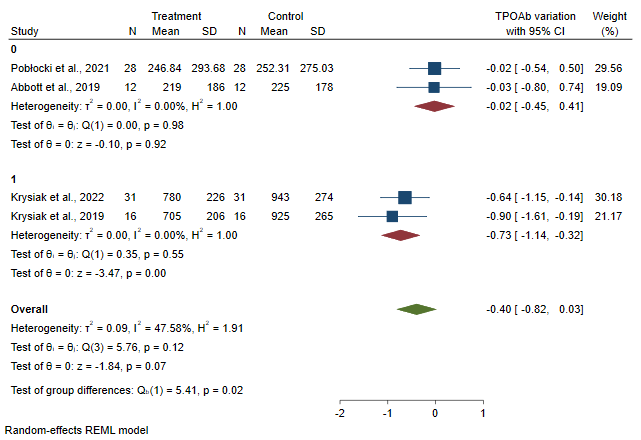


**Supplementary Figure 2.** Forest plot TPOAb sub-analysis. Legend: Any square identifies the weight of the study. The diamond represents the pooled result and its wideness indicates 95%CI. 0: Studies including patients with HT. 1: Studies including patients with HT and GRC.


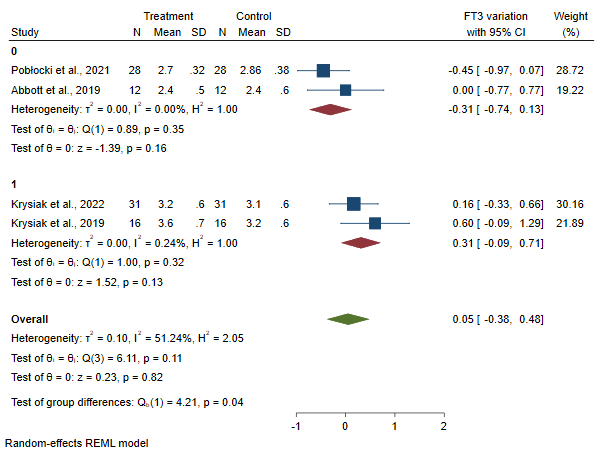


**Supplementary Figure 3.** Forest plot FT3 sub-analysis. Legend: Any square identifies the weight of the study. The diamond represents the pooled result and its wideness indicates 95%CI. 0: Studies including patients with HT. 1: Studies including patients with HT and GRC.
